# Oxaliplatin, 5-fluorouracil/leucovorin and epirubicin as first-line treatment in advanced gastric carcinoma: a phase II study

**DOI:** 10.1038/sj.bjc.6603644

**Published:** 2007-03-13

**Authors:** B Neri, P Pantaleo, E Giommoni, R Grifoni, C Paoletti, V Rotella, D Pantalone, A Taddei, A Mercatelli, P Tonelli

**Affiliations:** 1 Department of Oncology, Centre of Experimental and Clinical Oncology, University of Florence, Florence, Italy; 2 Unit of General Surgery, Department of Critical Medicine, University of Florence, Florence, Italy; 3 Unit of General Surgery, ‘Azienda Ospedaliero-Universitaria Careggi’, Florence, Italy

**Keywords:** chemotherapy, epirubicin, 5-fluorouracil, gastric cancer, leucovorin, oxaliplatin

## Abstract

The association between oxaliplatin and 5-fluorouracil (5-FU) has been extensively reported to improve prognosis of gastric cancer patients. The present study is aimed at evaluating response rate and the toxicity profile of the association with oxaliplatin, 5-FU/lecovorin and epirubicin in gastric cancer patients with locally advanced or metastatic disease. Thirty-six patients have been enrolled and 35 evaluated. The treatment schedule was oxaliplatin (100 mg m^−2^), 5-FU (400 mg m^−2^), leucovorin (40 mg m^−2^) and epirubicin (60 mg m^−2^) intravenously. administered every 3 weeks for 6 months, for a total of 185 therapy cycles . Response rate and toxicity were assessed according to the international WHO criteria. Every patient received a mean of 5.3 therapy cycles in a day-hospital setting. Sixteen of 35 patients (46%) showed an objective response, two complete response and 14 partial response. Median time to progression was 33 weeks with an overall median survival of 49 weeks. During the study, anaemia grade 3 and neutropenia grade 3 were observed in 9 and 11% of patients respectively. A grade 3 periferic sensorial neuropathy was observed in 6% of patients. No life threatening or cardiac toxicity was recorded. The regimen used showed anticancer activity against gastric carcinoma, a tolerable toxicity profile and excellent patient compliance.

Gastric cancer is the second most frequent tumour worldwide and one of the most frequent causes of cancer-related deaths. Although the incidence and mortality for gastric cancer are decreasing in many countries and in spite of important changes in the therapeutic options over the last decade, gastric cancer still represents the second most frequent malignancy in the world and the fourth in Europe (Parkin *et al*, 2001), with a poor prognosis even at an early stage of the disease. Also, patients with gastric cancer typically present with advanced disease. For patients presenting with an earlier stage of disease, more than 50% undergo surgery, but even after a curative resection, 60% eventually relapse locally or with distant metastases with a median survival time of 4–6 months, that may improve up to 7–10 months with a chemotherapy regimen. Although early diagnosis is the mainstay of successful treatment, even if radically treated, about 50% of patients present a disease relapse or spread within 5 years from diagnosis (Wilke *et al*, 1991; Wagner *et al*, 2005). Hence, in locally advanced or metastatic disease, the only available choice appears to be systemic chemotherapy (Macdonald *et al*, 1979; Preusser *et al*, 1989; Wagner *et al*, 2005), that has proved to improve survival compared with supportive care alone (hazard ratio (HR): 0.39; 95% confidence interval (CI): 0.28–0.52). Combination therapy regimens were found more effective than single-agent therapies, particularly 5-fluorouracil (5-FU), (HR: 0.85; 95% CI:0.76–0.96). Among polychemotherapies those containing the combination with cisplatin (DDP), 5-FU and antracycline derivatives proved more effective than those without antracyclines (HR: 0.77; 95% CI: 0.62–0.95) suggesting that polychemotherapy may significantly improve survival among patients with advanced stage gastric cancer. Recognition of epirubicin's (EPI) activity in gastric cancer and its better toxicity profile, compared with doxorubicin (Launchbury and Habboubi, 1993), have led to incorporate it into combination regimens for advanced gastric cancer. In the last decade, oxaliplatin (L-OHP), a third-generation platinum derivative, has been found to represent an intriguing alternative to cisplatin, as it shows a comparable activity but a more favourable global toxicity profile (Misset *et al*, 2000). Moreover, because 5-FU is considered a cornerstone of therapy for gastric cancer, combining it with L-OHP is logical, and there is considerable evidence of preclinical synergy between the two agents (Raymond *et al*, 1998). Clinical studies with L-OHP–5FU-based regimen reported a high response rate ranging from 43 to 55% in the treatment of gastric cancer and an excellent toxicity profile (Al Batran *et al*, 2004; De Vita *et al*, 2005).

On the basis of these data, we designed this phase II study to determine the response rate and toxicity profile of the combination chemotherapy of L-OHP–5FU/LV–EPI as first-line treatment in locally advanced and/or metastatic gastric cancer.

## PATIENTS AND METHODS

### Eligibility

From December 2001 to November 2003, 36 patients with histologically proven gastric cancer, locally unrecsectable, recurrent after surgery or with metastatic localisations entered the study, after being thoroughly informed of the study design, benefits and risks according to the guidelines of local ethic committee and the Declaration of Helsinki Principles, and all of them signed their consent. All patients had measurable disease and met the following criteria: performance status (ECOG)⩽2, life expectancy of at least 3 months and age ⩽75 years . Laboratory acceptance parameters included a white blood cell count above 4000 cells *μ*l^−1^, a haemoglobin level not lower than 9.5 g dl^−1^, a platelet count not less than 100 000 *μ*l^−1^, serum transaminase <3 × the upper normal limit (UNL) and bilirubin and creatinine values of <1.5 × UNL. Contraindication to entry included an active infectious process, an active heart disease, central nervous system involvement or any concomitant second primary cancer. Patients who had received previous chemotherapy were also excluded.

### Pretreatment evaluation

History, full-body examination and tumour-related symptoms were recorded for each patient. Further assessment included vital sign examination, performance status determination and laboratory tests (haematology, blood chemistry and urinalysis). At study entry the following investigations were obligatory performed: bone scintigraphy, ECG, echocardiogram with evaluation of ventricular function, chest X-ray, abdominal ultrasound, computed tomography (CT) and, only if indicated, CT scan of the thorax.

### Treatment schedule and dose modifications

Oxaliplatin (100 mg m^−2^), 5-FU (400 mg m^−2^), LV (40 mg m^−2^) and EPI (60 mg m^−2^) were administered intravenously, on an outpatient basis. The treatment was repeated every 3 weeks or until evidence of disease progression, patient refusal or unacceptable adverse reactions. The overall treatment was programmed over a 6 month period (maximum eight cycles), considering that the risk of developing severe disturbance of neurologic function is related to L-OHP neurotoxicity, that generally becomes a clinical problem when the cumulative dose approximates 800 mg m^−2^ (Extra *et al*, 1998). Full doses of the anticancer drugs were given if granulocyte count was >1500 *μ*l and platelet count was >100 000 *μ*l. In the case of grade 2 or more toxicity excepted alopecia, chemotherapy was discontinued for a week and than restarted after full recovery. During the study, leukocite-stimulating growth factors were allowed in patients showing grade 3 or more neutropenia. Reduction of 25% in all the drugs dose was performed in the event of a second consecutive occurrence of grade >2 toxicity. Patients with unsolved grade 2 or more toxicity after two consecutive treatment delays or experiencing grade 3 and 4 nonhaematological toxicity except alopecia went off study.

### Treatment response and toxicity

For evaluation of tumour response, the best objective imaging technique for the each individual patient was decided following pretreatment evaluation (either CT scan, ultrasound or conventional X-ray imaging). The same technique was then performed every 6 weeks by the same investigator and tumour response was assessed together with a second independent radiologist.

All the patients were thereafter examined the 15th day of every cycle and before the start of every following cycle. Toxicity and response rate were assessed and evaluated according to the WHO criteria (1979).

### Statistical methods

According to the optional Simon two-step design, if a minimum objective response rate >40% was observed in the first 15 patients, an additional 15 patients were enrolled and if >12 responses were observed in 30 patients (40%), the regimen was considered active and submitted for further evaluation (Simon, 1989). The descriptive statistics were reported as proportions and medians. Time to disease progression (TTP), was defined as the interval between initial treatment and the time of disease progression or death. Survival time was calculated from the date of treatment initiation until the date of the last follow-up evaluation or death. TTP and overall survival (OS) were analysed according to the Kaplan–Meier method (Kaplan and Meier, 1958). The CIs for response rates, TTP and OS were calculated using methods for exact binominal CI (Lentner, 1982) . Survivors were censored on the last date they were known to be alive.

## RESULTS

From December 2001 to November 2003 a total of 36 patients (25 male, 10 female patients, age range 39–72 years, mean age 58 years) were enrolled. Among them, one patient denied consent to undergo the second treatment and dropped out from the study. Among the 35 patients who completed the study and were evaluated for the above mentioned parameters, 27 (77%) had a performance status up to 1. Twenty-four of 35 had undergone surgery before the study. The large majority of patients had a metastatic disease (21/36; 58%), with a high prevalence of nodal or hepatic disease spread (Table 1).

Patients received a total amount of 185 therapy cycles (5.3 cycles/patient, range 2–8). At the end of the study, two patients experienced complete response (6%) and 14 partial response (40%). The overall response rate was 46% (95% CI: 30.2–63.8), with a median disease-free survival time of 27 weeks (95% CI: 20–31; range 7–104+).

**Figure 1:**
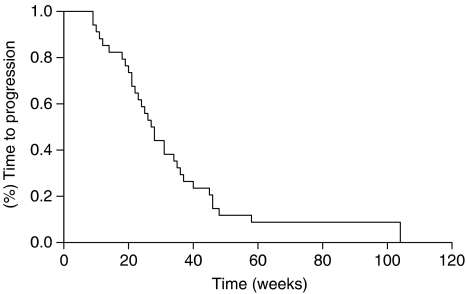
Kaplan–Meier estimates of time to progression among patients with gastric cancer treated with oxaliplatin, 5-fluorouracil/leucovorin and epirubicin regimen.

**Figure 2:**
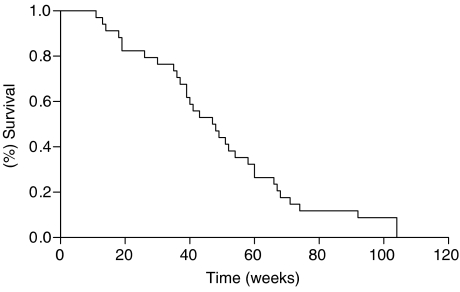
Kaplan–Meier estimates of OS among patients with gastric cancer treated with oxaliplatin, 5-fluorouracil/leucovorin and epirubicin regimen.

Among the other patients, 13 patients showed a stable disease (37%) whereas six patients presented disease progression (17%). The median time to disease progression was 33 weeks (95% CI: 17–49; range 6–104+) with a median survival time of 49 weeks (95% CI: 32—66; range 9–104+) (Table 2, Figures 1 and 2). The overall toxicity is depicted in Table 3. The most frequent toxic therapy effects were haematological (grade 3 toxicity in seven patients, anaemia in three and neutropenia in four.) Severe diarrhoea was present in 2 patients. No grade 4 toxicity was reported. The L-OHP-related peripheral neuropathy appeared to be mild and reversible in the majority of cases. No severe cardiac toxicity or death among the patients was recorded during the study.

## DISCUSSION

Gastric cancer remains a major cause of cancer death worldwide, with a very poor prognosis in patients with advanced disease. Even though chemotherapy is found to be more beneficial than supportive care alone in patients with advanced or metastatic disease, a standard chemotherapy regimen for gastric cancer has not yet been established. Among antiblastic agents, 5-FU is widely used, but the poor response in patients with advanced stage disease suggests its use it in association with other antiblastic agents. Several combination therapies that have reported satisfactory results in a phase II study were not confirmed in a randomised controlled context. Recently, L-OHP, a third-generation DDP-derived molecule, showed to be effective in the treatment of disease relapse after 5-FU treatment (Machover *et al*, 1996) with a reported response rate in gastro-intestinal tract malignancies ranging from 10 to 20%. The combination of L-OHP with 5-FU has been found to be effective in advanced stage with a mild to moderate toxicity (De Vita *et al*, 2005; Lordick *et al*, 2005). Moreover, the use of L-OHP may be helpful in overcoming drug resistance-related therapy drawbacks as its molecule does not share the same action mechanism of DDP.

In the present study, we administered in a 3-week-based regimen the combination of L-OHP–5FU/LV–EPI as first-line therapy to patients with locally advanced, recurrent or metastatic gastric cancer. The overall response rate in our patient group was high, as 16 out of the 35 patients that completed the study had an objective response (46%), and two of them had a complete response (6%). The median time to progression was 33 weeks (8.2 months), with an OS of 49 weeks (12.2 months). The high median survival time did not appear correlated with *a priori* criteria in patient selection, as most patients (58%) had a metastatic disease at the beginning of the study and 60% of these had multiple metastatic localisations. The results of recent studies using different DDP- or L-OHP-based regimens reported in Table 4 show that efficacy and tolerability are better than in other studies using anthracyclines (Macdonald *et al*, 1979; Preusser *et al*, 1989; In Woo *et al*, 2005). The L-OHP–5FU/LV–EPI regimen was better tolerated even when compared with other treatment schedules in which 5-FU is used as a single agent or in combination (Lordick *et al*, 2005). Haematological toxicity was characterised by grade 3 anaemia and neutropaenia and was present in 9 and 11% of patients, respectively. As L-OHP-related acute peripheral neuropathy was generally mild, short-lived and completely reversible within a few hours, treatment was not discontinued. Cumulative sensory neurotoxicity was present only in 6% of patients. Furthermore, L-OHP-induced neurotoxicity was reversible, with a median time to recovery of 12 weeks from grade 3 toxicity. As previously reported by De Vita *et al* (2005), this study confirms that the low incidence of cumulative sensory neurotoxicity could be related to the rather low L-OHP median cumulative dose received by each patient (530 mg m^−2^).

The current study suggests that the combination therapy L-OHP–5FU/LV–EPI is an active regimen in advanced gastric cancer that has the advantage of being well-tolerated and more easily administered in an outpatients setting. Further randomised controlled studies are mandatory to compare efficacy of this therapy with other association therapies. We do believe that the association L-OHP–5FU/LV–EPI could improve prognosis and the survival of patients with advanced stage gastric cancer.

## Figures and Tables

**Table 1:** Patient characteristics

	**No. of patients**
Enrolled/evaluable	36/35
	
*Gender*	
Male	25 (70%)
Female	11 (30%)
	
*Age*	
Mean	58 years
Range	39–72 years
	
*Performance status*	
Grade 0	9 (25%)
Grade 1	20 (56%)
Grade 2	6 (17%)
	
*Disease stage*	
Locally advanced or recurrent	15 (42%)
Metastatic	21 (58%)
	
Number of metastatic sites	
1	10 (50%)
2	7 (33%)
3	4 (17%)
	
*Metastasis localisation*	
Liver	12
Nodes	9
Peritoneal	8
Lung	2
Bone	4
Ovarian	1

**Table 2:** Response to treatment

**Evaluable patients no.**	**35**
Number of cycles	185
Mean cycles/patient	5.3
Range	2–7
CR	2 (6%)
PR	14 (40%)
Stable Disease:	13 (37%)
Progression of disease	6 (17%)
Overall response rate (CR+PR)	16 (46%)
Median time to progression (weeks)	33 (6–104+)
Median response duration (weeks)	27 (7–104+)
Median survival (weeks)	49 (9–104+)

CR=complete response; PR=partial response.

**Table 3:** Toxicities

**WHO Grade**	**1**	**2**	**3**	**4**
Anaemia	22 (62%)	10 (29%)	3 (9%)	—
Neutropaenia	17 (49%)	14 (40%)	4 (11%)	—
Thrombocitopenia	23 (65%)	11 (32%)	1 (3%)	—
Anorexia	24 (68%)	11 (32%)	—	—
Nausea	22 (62%)	13 (38%)	—	—
Vomiting	21 (60%)	14 (40%)	—	—
Diarrhoea	19 (54%)	14 (40%)	2 (6%)	—
Stomatitis	24 (68%)	11 (32%)	—	—
Neurotoxicity	20 (56%)	13 (38%)	2 (6%)	—

**Table 4:** Results of DDP- or L-OHP-based regimens in advanced gastric cancer

**Study**	**No. patients**	**Regimen**	**Overall response rate (%)**	**Median TTP (months)**	**Median OS (months)**
Al Batran *et al* (2004), *J Clin Oncol*	41	L-OHP/5-FU	43	5.6	9.6
De Vita *et al* (2005), *Br J Cancer*	61	L-OHP/5-FU	45	7.1	11.2
Lordick *et al* (2005), *Br J Cancer*	48	L-OHP/5-FU	48	6.5	11.4
		Leucovorin			
Thuss-Patience *et al* (2005), *J Clin Oncol*	45	EPI/DDP/5-FU	37	5.5	9.5
	45	Docetaxel/5-FU	35	5.3	9.7
Woo *et al* (2005), *Jpn J Clin Oncol*	35	EPI/DDP/tegafur	41	5.1	8.1
Lee *et al* (2007), *Ann Oncol*	48	L-OHP/CPT-11/5-FU	66	9.6	14.8
		Leucovorin			
Park *et al* (2006), *Br J Cancer*	20	L-OHP/capecitabine	65	7.5	—

DPP=cisplatin; EPI=epirubicin; 5-FU=5-fluorouracil; L-OHP=oxaliplatin.
